# Interferon-γ Release Assays for the Diagnosis of Tuberculosis and Tuberculosis Infection in HIV-Infected Adults: A Systematic Review and Meta-Analysis

**DOI:** 10.1371/journal.pone.0032482

**Published:** 2012-03-05

**Authors:** Miguel Santin, Laura Muñoz, David Rigau

**Affiliations:** 1 Department of Infectious Diseases, Bellvitge University Hospital-IDIBELL, L'Hospitalet, Barcelona, Spain; 2 University of Barcelona, Barcelona, Spain; 3 Spanish Network for Research in Infectious Diseases (REIPI), Instituto de Salud Carlos III, Madrid, Spain; 4 Iberoamerican Cochrane Center, Barcelona, Spain; McGill University, Canada

## Abstract

**Background:**

Despite the widespread use of interferon-γ release assays (IGRAs), their role in diagnosing tuberculosis and targeting preventive therapy in HIV-infected patients remains unclear. We conducted a comprehensive systematic review to contribute to the evidence-based practice in HIV-infected people.

**Methodology/Principal Findings:**

We searched MEDLINE, Cochrane, and Biomedicine databases to identify articles published between January 2005 and July 2011 that assessed QuantiFERON®-TB Gold In-Tube (QFT-GIT) and T-SPOT®.TB (T-SPOT.TB) in HIV-infected adults. We assessed their accuracy for the diagnosis of tuberculosis and incident active tuberculosis, and the proportion of indeterminate results. The search identified 38 evaluable studies covering a total of 6514 HIV-infected participants. The pooled sensitivity and specificity for tuberculosis were 61% and 72% for QFT-GIT, and 65% and 70% for T-SPOT.TB. The cumulative incidence of subsequent active tuberculosis was 8.3% for QFT-GIT and 10% for T-SPOT.TB in patients tested positive (one study each), and 0% for QFT-GIT (two studies) and T-SPOT.TB (one study) respectively in those tested negative. Pooled indeterminate rates were 8.2% for QFT-GIT and 5.9% for T-SPOT.TB. Rates were higher in high burden settings (12.0% for QFT-GIT and 7.7% for T-SPOT.TB) than in low-intermediate burden settings (3.9% for QFT-GIT and 4.3% for T-SPOT.TB). They were also higher in patients with CD4^+^ T-cell count <200 (11.6% for QFT-GIT and 11.4% for T-SPOT.TB) than in those with CD4^+^ T-cell count ≥200 (3.1% for QFT-GIT and 7.9% for T-SPOT.TB).

**Conclusions/Significance:**

IGRAs have suboptimal accuracy for confirming or ruling out active tuberculosis disease in HIV-infected adults. While their predictive value for incident active tuberculosis is modest, a negative QFT-GIT implies a very low short- to medium-term risk. Identifying the factors associated with indeterminate results will help to optimize the use of IGRAs in clinical practice, particularly in resource-limited countries with a high prevalence of HIV-coinfection.

## Introduction

Tuberculosis is one of the leading causes of mortality in people living with human immunodeficiency virus (HIV) worldwide, particularly in sub-Saharan Africa, where it is responsible for up to half of HIV-related deaths [1], [2].

HIV co-infection increases the risk of tuberculosis either by facilitating reactivation of a remote latent infection (LTBI) or by favoring the progression of a recently acquired infection towards active disease. Therefore, rapid identification and early treatment of active tuberculosis cases in order to interrupt further transmission, as well as the detection and treatment of LTBI to prevent progression to active disease, are crucial for controlling HIV-associated tuberculosis [3]. However, the lack of accuracy of clinical and radiographic manifestations of tuberculosis in HIV-infected patients and the limitations of diagnostic tests pose great obstacles to rapid diagnosis and delay the initiation of specific treatment [4]. Furthermore, the well-known shortcomings of the tuberculin skin test (TST) for diagnosing LTBI hamper the accurate targeting of HIV-infected patients for isoniazid preventive therapy (IPT) [5].

T-cell-based interferon-gamma (IFN-γ) release assays (IGRAs) constitute a promising alternative to TST for diagnosing tuberculosis infection. IGRAs use highly *M. tuberculosis*-specific antigens which are not present in most non-tuberculous mycobacteria or in the bacillus Calmette-Guérin vaccine [6]. Two commercial tests are available: the QuantiFERON®-TB Gold In-Tube (QFT-GIT) test (Cellestis Ltd, Carnegie, Australia), which uses ELISA to detect IFN-γ in the culture supernatant, and the T-SPOT®.TB (Oxford Immunotec, Abingdon, UK), which is based on the enzyme-linked immunospot (ELISpot) assay.

In low-burden tuberculosis settings, IGRAs have shown better specificity and equal or greater sensitivity than TST for the detection of tuberculosis infection, and a better correlation with the intensity of exposure to a source of infection [7]–[9]. These advantages have raised great hopes for a better assessment of tuberculosis infection in people at risk, particularly in immunosuppressed and BCG-vaccinated individuals. Although in the absence of any supporting evidence, IGRAs have also been increasingly used as diagnostic tests for active tuberculosis. This practice has raised concern, particularly in high-burden and resource-limited countries, where the high background LTBI prevalence and the HIV-associated immunosuppression may limit their potential value as rule-in or rule-out tests. Based on recently published meta-analyses showing a suboptimal accuracy for either diagnosing or predicting subsequent active tuberculosis [10]–[12], the World Health Organization (WHO) issued a consensus statement in which an expert panel advised against the use of IGRAs for diagnosing active tuberculosis, irrespective of HIV status, or for identifying people at risk for active tuberculosis disease in low- and middle-income countries [13]. With regard to HIV-infected people, the WHO report stressed the very low quality of evidence for using IGRAs in these patients, and recommended that these tests should not replace TST for the assessment of LTBI [13].

Although IGRAs were not developed to replace conventional microbiological methods for the diagnosis of active tuberculosis disease, they may have an adjunctive role in symptomatic patients with suspicion of active disease by complementing clinical-radiographic and epidemiological data to guide diagnosis work-up. Therefore, knowing how HIV infection compromises the IGRAs' ability to detect tuberculosis infection in patients with active disease is essential in order to determine their role in different clinical and epidemiological settings.

We conducted a comprehensive systematic review (SR) to assess the sensitivity and specificity of IGRAs for the diagnosis of active tuberculosis disease, their value to predict development of subsequent active tuberculosis, and the proportion of indeterminate results in HIV-infected adults. Whenever feasible, we assessed how HIV-associated CD4^+^ T-cell depletion affects IGRA performance, and tried to identify differences according to tuberculosis burden settings and HIV infection status.

## Methods

This SR was conducted in accordance with the PRISMA statement [14]. Ethical approval was not required for this study.

### Search

We systematically searched for studies published between 1 January 2005 and 31 July 2011 that evaluated the diagnostic performance of IGRAs for tuberculosis or LTBI in HIV-positive adult populations (or populations with at least five HIV-positive individuals). We searched MEDLINE, the Cochrane Central Register of Controlled Trials, and the Biomedicine Database (IME) of the Spanish National Research Council (CSIC). Searches comprised a combination of the following terms: “HIV”, “immunosuppressed patients”, “tuberculosis”, “latent tuberculosis infection”, “QuantiFERON”, “QuantiFERON-TB Gold”, “T-SPOT.TB”, “interferon-gamma release assays”, and “T-cell assays”, as listed in titles, abstracts or text words. Searches were limited to studies published in English or Spanish. We also reviewed citations of the original and review articles, and guidelines for additional references. When necessary, we contacted the authors of the studies for additional information.

### Selection

For our analysis, we selected only prospective studies that usedthe commercial tests QuantiFERON®-TB Gold In-Tube and T-SPOT®.TB performed in blood with 16–24 h of incubation. We excluded studies of non-commercial IGRAs or studies based on the old version of the ELISA assay (QuantiFERON®-TB Gold), as well as studies presenting non-original data, conference abstracts, editorials, reviews, guidelines, and studies conducted in animals.

### Quality assessment

We checked the quality of the studies used to calculate assay accuracy with the QUADAS check list [15].

In the case of indeterminate results, we appraised the quality of the studies by assessing whether or not a definition was given in the methods section (“performed and interpreted according to the manufacturer's instructions” was acceptable), and whether or not data for the two types of indeterminate tests (low IFN-γ production in the positive control or high IFN-γ production in the negative control) were reported separately. In addition, since an insufficient number of peripheral blood mononuclear cells (PBMCs) precludes performance of the T-SPOT.TB test, we also checked whether or not these unsuccessful test attempts (failure tests) had been reported.

To evaluate the quality of the studies that assessed the risk of subsequent tuberculosis according to the result of an IGRA assay, we used the Newcastle-Ottawa Scale (NOS) for non-randomized cohort studies [16].

### Data extraction

Two researchers (M.S. and L.M.) independently compiled the data using a standardized data extraction sheet. Discrepancies were resolved by discussion and consensus. The following data were extracted: year of publication, period and country, number of participants, gender, test evaluated, CD4^+^ cell count, development of active tuberculosis, TST results, indeterminate test results (overall and by two CD4^+^ cell count thresholds) and fraction of individuals with true positive, false negative, true negative and false positive results for the calculation of the test sensitivity and specificity.

### Quantitative data synthesis and analysis

We assessed the following outcomes for each study and pooled them when feasible: sensitivity and specificity for active tuberculosis, predictive value for incident active tuberculosis, and rates of indeterminate results. The following definitions were used: sensitivity refers to the proportion of culture-proven tuberculosis patients who had a positive IGRA test, and specificity refers to the proportion of symptomatic non-tuberculosis patients who had a negative IGRA test. For the sensitivity calculation, we included only patients with confirmed tuberculosis (either with a positive culture for *M. tuberculosis*, a positive nucleic acid amplification test, or characteristic histopathological findings and response to specific treatment) that was still untreated or had been treated for less than two weeks. Indeterminate results were included as false negatives. For the specificity calculation, we selected studies that had enrolled patients with suspected active tuberculosis (either symptoms potentially caused by tuberculosis or a clinical and radiographic picture suggestive of tuberculosis). Results due to low IFN-γ production in the phytohaemagglutinin (PHA)-stimulated well or high background IFN-γ production were defined as indeterminate. Since T-SPOT.TB tests not performed due to insufficient PBMC cells are usually excluded from the analyses, we used the term “failure tests” for unrealizable tests. We assessed the effect of immunosuppression on sensitivity and indeterminate result rates by pooling and stratifying the results for 200 CD4^+^ T-cell count threshold.

Results are presented for each IGRA assay and for countries grouped by tuberculosis burden: high-burden (>40 cases per 100,000 population), low- to intermediate-burden (<40 cases per 100,000 population) [17]. Head-to-head comparisons between HIV-infected and HIV-uninfected individuals, as well as between the two IGRA assays and TST, were performed whenever possible.

We calculated combined estimates of pooled sensitivity, specificity and the 95% confidence interval (CI). The pooled effect for binary outcomes was presented as the difference with the 95% CI. A random-effects synthesis model meta-analysis was used to pool the effect across the studies. Inconsistency was quantified by the *I* ^2^ statistic. Forest plots were constructed to show the effect size of all the studies and the variability of the pooled estimates. The analyses were performed with MetaAnalyst software [18].

## Results

### Characteristics of the studies

**Figure 1:**
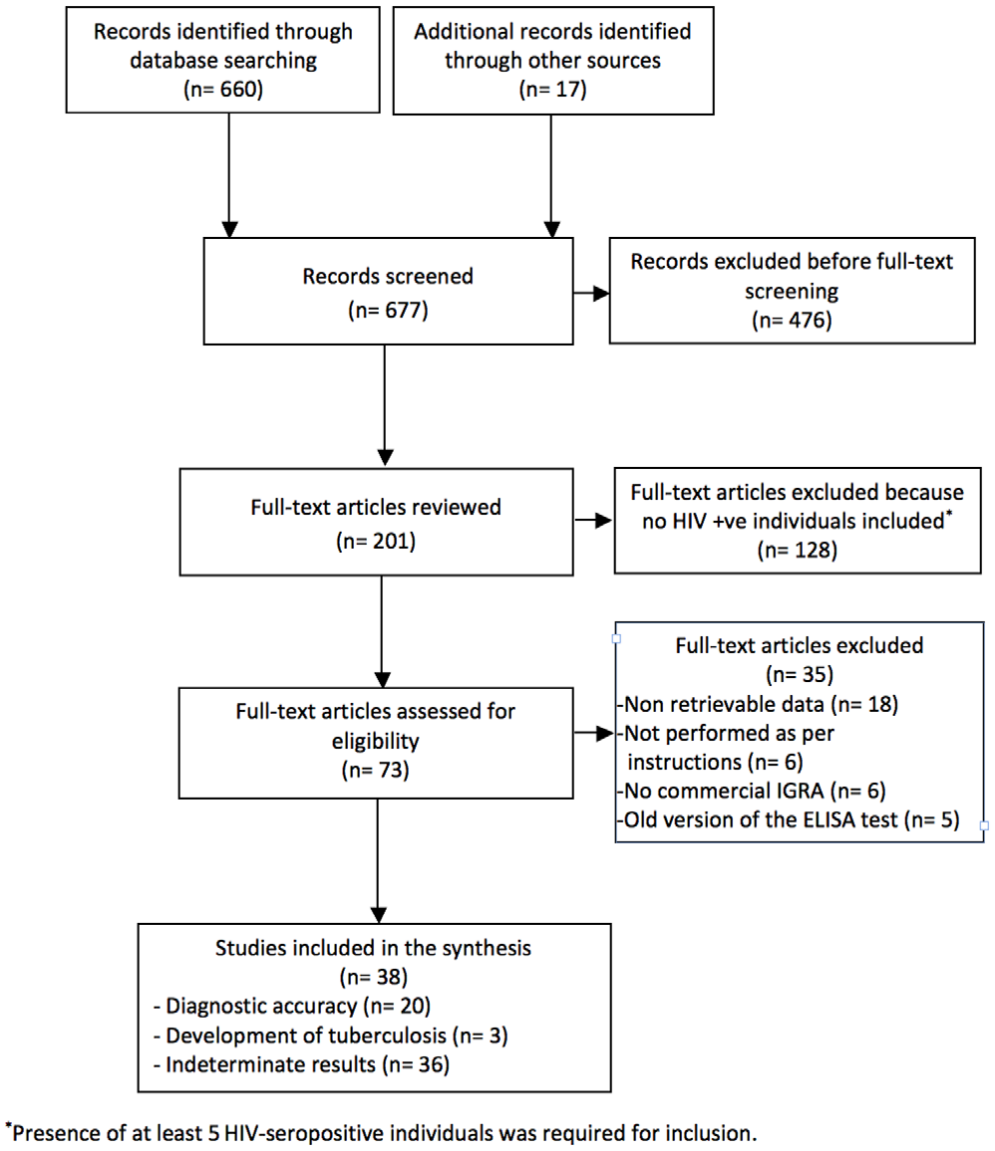
Flowchart for study selection.

Of the total of 677 citations identified, 38 were eligible for analysis (Figure 1) [19]–[56]. The studies included were conducted in 17 different countries, 19 (50%) in high-burden countries, 18 (47.4%) in low-burden tuberculosis countries, and one (2.6%) included participants from both settings. Thirty-seven were published in English and one in Spanish. Some industry involvement was reported in 11 studies (28.9%), mainly in the form of donation of IGRA kits to the researchers. Of the 20 studies used to calculate sensitivity/specificity, 13 (65%) corresponded to high-burden countries. A summary of the 38 studies included is provided in Table 1. Detailed information on the studies included in the review is available upon request.

**Table 1:** General and outcome-related characteristics of the 38 studies.

		Outcome		
Variable	Overall	Diagnosis of active tuberculosis	Development of tuberculosis	Indeterminate results
**Studies**	38	20	3	36
**Tuberculosis burden setting**				
High-burden countries	19	13	–	18
Low/intermediate-burden countries	18	7	3	17
Both*	1	–	–	1
**Individuals enrolled**				
Total				
-HIV-infected	6514	3155	1166	6434
-HIV-uninfected	3437	1034	135	3437
Median (IQR)				
-HIV-infected	90 (160)	98 (138)	266 (362)	107 (166)
-HIV-uninfected	106 (194)**	106 (180)	–	105 (178)
**Male : female ratio** †	2.8 : 1	1.5 : 1	2.3 : 1	2.6 : 1
**CD4+ counts, cells/µL** ‡				
Median (16 studies), n/N (%)				
<200	7/18 (39)	7/8 (88)	2/3 (67)	12/16 (75)
<350	12/18 (67)	7/8 (88)	2/3 (67)	12/16 (75)
**Test evaluated**				
QFT-GIT	17	10	2	17
T-SPOT.TB	11	5	1	17
QFT-GIT & T-SPOT.TB	10	5	0	9

IQR: interquartile range; n/N: number of studies with the condition/total number of studies;

* Switzerland and sub-Saharan area.

** Calculated from 17 studies enrolling HIV-uninfected individuals.

† Data available for 34 studies;

‡ Only HIV-infected individuals.

### Quality of the studies

Eight of the 20 studies (40%) used to calculate sensitivity and specificity met all the quality indicators, ten (50%) met between 75% and 100%, and two (10%) met less than 75%.

Indeterminate results due to high production of IFN-γ in the negative control were either not defined as such or excluded from the analysis in 32% of studies with QFT-GIT and in 28% with T-SPOT.TB. The results for the two types of indeterminate results were reported separately in 27% of studies with QFT-GIT and in 32% with T-SPOT.TB. Only three studies (16%) provided data on the T-SPOT.TB tests not performed because of insufficient quantities of cells.

As for the studies evaluating the ability of IGRAs to predict subsequent tuberculosis, follow-up was adequate in all three (12, 19 and 20 months respectively), and the exposed sample was representative of the HIV population; however, there was no adequate outcome assessment (pre-test and during follow-up) in any of them, and the number of incident tuberculosis cases was low (zero, two and three cases respectively). More detailed information on the quality of the studies is available upon request.

### Sensitivity and specificity for active tuberculosis

#### Sensitivity

**Figure 2:**
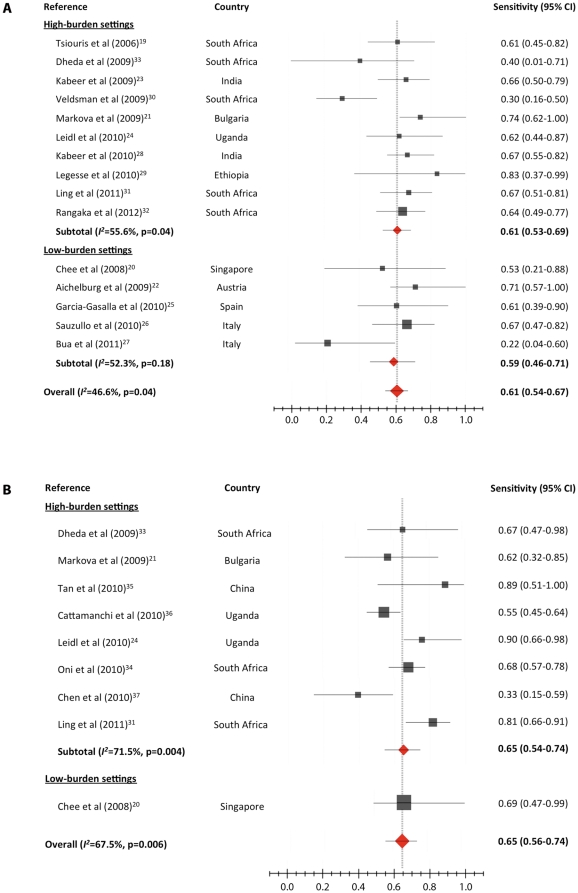
Sensitivity of QuantiFERON-TB Gold In-Tube (A) and T.SPOT.TB (B), in HIV-infected patients with confirmed tuberculosis, stratified for tuberculosis burden setting. Pooled estimates derived from random effects (DerSimonian-Laird) modeling.

The sensitivity of QFT-GIT was estimated from 15 studies with a total of 356 participants with culture-proven active tuberculosis [19]–[33], and the sensitivity of T-SPOT.TB was estimated from nine studies with 311 participants with culture-proven active tuberculosis [20], [21], [24], [31], [33]–[37]. The pooled sensitivity was 61% (95% CI 54–67; *I^2^* = 46.6%) for QFT-GIT and 65% (95% CI 56–74; *I^2^* = 67.5%) for T-SPOT.TB (Figure 2).

Five studies compared the sensitivity of QFT-GIT and T-SPOT.TB head to head [20], [21], [24], [31], [33]. The pooled sensitivity was 69% (95% CI 57–79; *I^2^* = 35.9) for QFT-GIT and 79% (95% CI 65–88; *I^2^* = 66.4) for T-SPOT.TB. The results of simultaneous TST and QFT-GIT in patients with active tuberculosis were reported in five studies [19], [22], [23], [25], [32]. The pooled sensitivity was 67% (95% CI 58–74; *I* ^2^ = 0.0%) for QFT-GIT and 60% (95% CI 34–82; *I* ^2^ = 46.2%) for TST. T-SPOT.TB was compared with TST in one study with 13 patients [38]: excluding the indeterminate results, the sensitivities were 85% and 46% for T-SPOT.TB and TST respectively. Tables 2 and 3 show the differences in sensitivity between QFT-GIT and T-SPOT.TB, and between IGRAs and TST respectively in HIV-infected patients.

**Table 2:** Head-to-head comparison of sensitivity between QFT-GIT and T-SPOT.TB in HIV-infected patients with culture-confirmed tuberculosis.

Reference	Country	Sensitivity QFT-GIT	Sensitivity T-SPOT.TB	Sensitivity difference
		n/N (%)	n/N (%)	QFT-GIT (%) – T-SPOT.TB (%)
Chee et al. [20]	Singapore	4/7 (57)	7/7 (100)	−43
Markova et al. [21]	Bulgaria	12/13 (92)	8/13 (62)	30
Leidl et al. [24]	Uganda	13/19 (68)	17/19 (89)	−21
Ling et al. [31]	South Africa	29/43 (67)	35/43 (81)	−14
Dheda et al. [33]	South Africa	1/5 (20)	5/5 (100)	−80

QFT-GIT: QuantiFERON®-TB Gold In-Tube; n/N: positive cases/cases with active tuberculosis.

**Table 3:** Head-to-head comparison of sensitivity between IGRAs and TST in HIV-infected patients with culture-confirmed tuberculosis.

Reference	Country	IGRA	Sensitivity IGRA	Sensitivity TST	Sensitivity difference
			n/N (%)	n/N (%)	IGRA (%) – TST(%)
Tsiouris et al. [19]	South Africa	QFT-GIT	17/26 (65)	22 (85)	−20
Aichelburg et al. [22]	Austria	QFT-GIT	10/11 (91)	8 (80)*	11
Kabeer et al. [23]	India	QFT-GIT	29/44 (66)	11 (25)	41
Garcia-Gasalla et al. [25]	Spain	QFT-GIT	9/13 (69)	5 (42)**	27
Rangaka et al. [32]	South Africa	QFT-GIT	32/50 (64)	34 (68)	−4
Vincenti et al. [38]	Italy	T-SPOT.TB	11/13 (85)	6 (46)†	39

IGRAs: Interferon-γ release assays; QFT-GIT: QuantiFERON®-TB Gold In-Tube; TST: Tuberculin skin test; n/N: positive cases/cases with active tuberculosis.

* 8 positive tests of 10 cases;

** 5 positive tests of 12 cases;

† Indeterminate results of T-SPOT.TB excluded.

Six studies, covering 634 participants with tuberculosis (113 HIV-infected and 521 HIV-uninfected) evaluated the sensitivity of QFT-GIT according to HIV status [19], [20], [25], [29], [31], [33]. The pooled sensitivity was 65% (95% CI 55–74; *I* ^2^ = 9.8%) and 79% (95% CI 75–83; *I* ^2^ = 24.9%) in HIV-infected and HIV-uninfected patients respectively. As for T-SPOT.TB, three studies compared sensitivity between HIV-infected and uninfected patients (55 HIV-infected and 364 HIV-uninfected) [20], [31], [33]. The pooled sensitivity was 75% (95% CI 64–84; *I* ^2^ = 39.1%) in HIV-infected and 90% (95% CI 84–94; *I* ^2^ = 74%) in HIV-uninfected patients. Table 4 shows the differences in sensitivity of the two IGRA assays according to HIV status.

**Table 4:** Comparison of sensitivity of IGRAs between HIV-infected and HIV-uninfected patients with culture-confirmed tuberculosis.

Reference	Country	Sensitivity in HIV-pos	Sensitivity in HIV-neg	Sensitivity difference
		n/N (%)	n/N (%)	HIV-pos (%) – HIV-neg (%)
**QFT-GIT**				
Tsiouris et al. [19]	South Africa	17/26 (65)	11/15 (73)	−8
Chee et al. [20]	Singapore	4/7 (57)	220/273 (81)	−24
Garcia-Gasalla et al. [25]	Spain	12/13 (92)	85/105 (81)	11
Legesse et al. [29]	Ethiopia	13/19 (68)	20/31 (65)	3
Ling et al. [31]	South Africa	29/43 (67)	67/82 (82)	−15
Dheda et al. [33]	South Africa	1/5 (20)	11/15 (73)	−53
**T-SPOT.TB**				
Chee et al. [20]	Singapore	7/7 (100)	247/267 (93)	7
Ling et al. [31]	South Africa	35/43 (81)	70/82 (85)	−4
Dheda et al. [33]	South Africa	5/5 (100)	14/15 (93)	7

IGRAs: Interferon-γ release assays; QFT-GIT: QuantiFERON®-TB Gold In-Tube; n/N: positive cases/cases with active tuberculosis.

The effect of CD4^+^ cell counts on sensitivity was evaluated in three studies with QFT-GIT [23], [28], [31] and three studies with T-SPOT.TB [31], [34], [37]. While one study on QFT-GIT reported a decrease in its sensitivity with fewer than 200 circulating CD4^+^ cells [23], another found no differences in CD4^+^ T-cell counts between patients with positive and negative results [28]. A third study found higher sensitivity in patients with <200 CD4^+^ cells (76%; 95% CI 53–92) than in those with ≥200 CD4^+^ cells (61%; 95% CI 36–83) [31]. None of the three studies assessing T-SPOT.TB reported a relationship between lower sensitivity and lower CD4^+^ T-cell counts [31], [34], [37]. In fact, as in the case of QFT-GIT, the sensitivity of T-SPOT.TB in one study was higher in patients with CD4^+^ cells <200 (90%; 95% CI 67–99) than in those with CD4^+^ cells ≥200 cells (78%; 95% CI 52–94) [31].

#### Specificity

**Figure 3:**
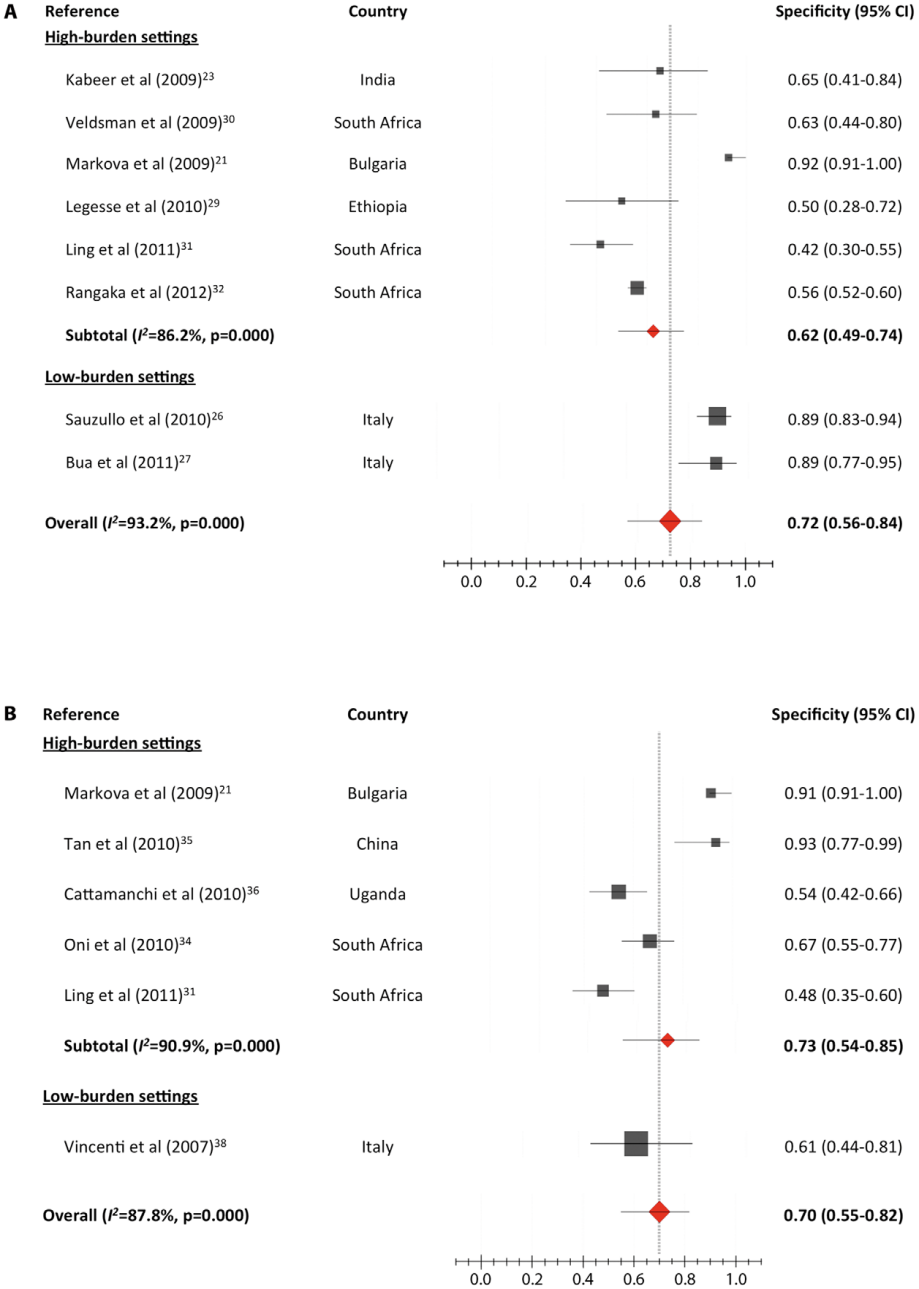
Specificity of QuantiFERON-TB Gold In-Tube (A) and T.SPOT.TB (B), in HIV-infected patients with confirmed tuberculosis, stratified for tuberculosis burden setting. Pooled estimates derived from random effects (DerSimonian-Laird) modeling.

The specificity of QFT-GIT was calculated from eight studies covering a total of 1334 participants without active tuberculosis [21], [23], [26], [27], [29]–[32], whilst that of T-SPOT.TB was calculated from six studies with a total of 326 participants without active tuberculosis [21], [31], [34]–[36], [38]. The pooled specificity was 72% (95% CI 56–84; *I^2^* = 93.2%) for QFT-GIT, and 70% (95% CI 55–83; *I^2^* = 87.8%) for T-SPOT.TB (Figure 3).

### Predictive value of IGRAs for incident active tuberculosis

Three longitudinal studies assessed incident active tuberculosis [22], [43], [52]; all of them were conducted in low-burden countries. In a prospective cohort study, 830 HIV-infected patients who underwent QFT-GIT testing were left untreated and were followed periodically [22]. Of 822 individuals without active tuberculosis at baseline, 36 were positive. After a median follow-up of 19 months, three (8.3%) patients with positive QFT-GIT developed tuberculosis, but none of the 705 patients with a negative QFT-GIT developed active disease. In another study with 201 HIV-seropositive individuals, two out of 20 infected patients with positive T-SPOT.TB who did not receive preventive treatment developed active tuberculosis during the first year [52]. In a third study assessing 135 HIV-infected individuals, none of the 103 patients who had a negative or indeterminate QFT-GIT result and negative TST at baseline developed tuberculosis after a median follow-up of 20 months [43] (Table 5).

**Table 5:** Risk of active tuberculosis according to IGRA result in patients not receiving preventive treatment.

Reference	Country	IGRA	No. of individuals	Median follow-up	Cumulative incidence	Risk difference IGRA-pos - IGRA-neg
				Median follow-up	Cases (%; 95%CI)	IGRA-pos - IGRA-neg % (95%CI)
Clark et al. [52]	U. Kingdom	T-SPOT.B	20 T-SPOT.TB-pos	12 months	2 (10%; 1.8–33.0)	10% (−3 to 23)
			114 T-SPOT.TB-neg	3 months	0 (0%; 0.0–3.0)	10% (−3 to 23)
Aichelburg et al. [22]	Austria	QFT-GIT	36 QFT-GIT-pos	19 months	3 (8.3%; 1.8–22.0)	8.3% (−0.7 to 17)
			705 QFT-GIT-neg		0 (0%; 0.0–0.5)	
			44 QFT-GIT Ind		0 (0%; 0.0–9.0)	
Santin et al. [43]	Spain	QFT-GIT	101 QFT-GIT-neg	20 months	0 (0%; 0.0–4.5)	N.A.
			2 QFT-GIT Ind			

QFT-GIT = QuantiFERON®-TB Gold In-tube; Ind. = indeterminate; N.A. = not applicable.

### Indeterminate IGRA results

**Figure 4:**
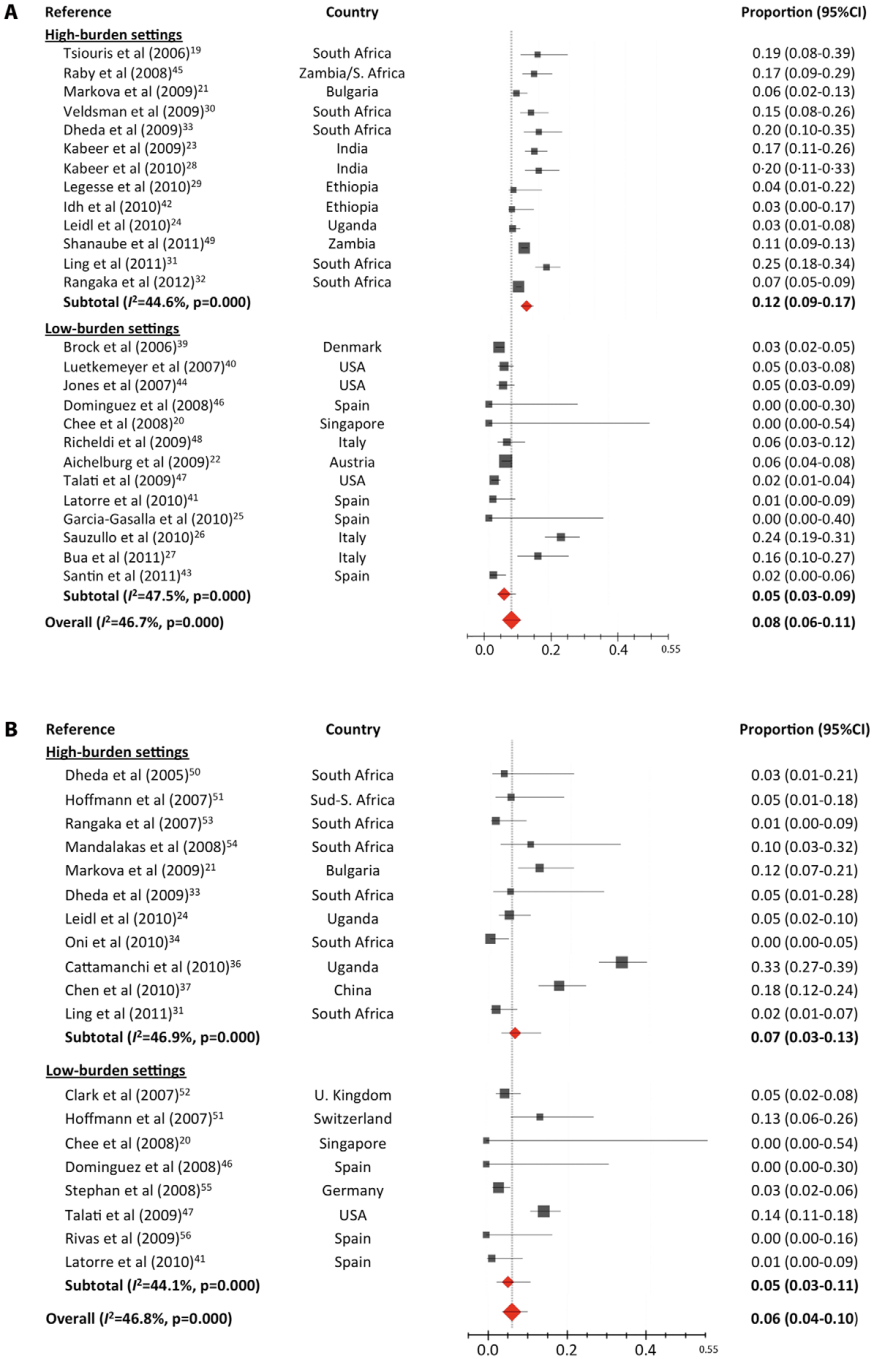
Proportion of indeterminate results of QuantiFERON-TB Gold In-Tube (A) and T-SPOT.TB (B) in HIV-infected patients, stratified for tuberculosis burden setting. Pooled estimates derived from random effects (DerSimonian-Laird) modeling.

Twenty-six studies reported data on indeterminate results in patients tested with QFT-GIT, with a total of 5209 participants [19]–[33], [39]–[49]. The pooled indeterminate rate was 8.2% (95% CI 6.0–11.2; *I* ^2^ = 46.8%) (Figure 4A). When the analysis was restricted to the seven studies that differentiated the two types of indeterminate results, 11 out of 120 (9.2%) indeterminate results were due to high background IFN-γ production (negative control), and the other 109 (90.8%) were due to low IFN-γ production in the positive control.

Eighteen studies including a total of 2236 participants reported data on indeterminate results with T-SPOT.TB [20], [21], [24], [31]–[34], [36], [37], [41], [46], [47], [50]–[56]. The pooled indeterminate rate was 5.9% (95% CI 3.5–9.8; *I* ^2^ = 47.0%) (Figure 4B). When the analysis was restricted to the three studies that provided data on failure tests (not performed due to an insufficient number of PBMCs), the pooled indeterminate rate was 20.6% (95% CI 11.1–34.9; *I* ^2^ = 48.3%). Of the 153 indeterminate results derived from these three studies, 51 (33.3%) were due to failure to perform the test, 50 (32.7%) were due to low IFN-γ production in the PHA-stimulated well, and 52 (34%) to high background IFN-γ production.

In eight studies that compared QFT-GIT and T-SPOT.TB head to head [20], [21], [24], [31], [33], [41], [46], [47]. Pooled proportion were 5.7% (95% CI 2.1–14.7; *I* ^2^ = 46.9%) for QFT-GIT, and 6.1% (95% CI 3.2–11.2; *I* ^2^ = 40.5%) for T-SPOT.TB (Table 6).

**Table 6:** Head-to-head comparison of the proportion of indeterminate results between QFT-GIT and T-SPOT.TB in HIV-infected patients.

Reference	Country	Population tested	Indeterminate results QFT-GIT	Indeterminate results T-SPOT.TB	Difference indeterminate results
			n/N (%)	n/N (%)	QFT-GIT(%) - T-SPOT.TB (%)
Chee et al. [20]	Singapore	Active TB	0/7	0/7	–
Markova et al. [21]	Bulgaria	Symptomatic ptes	5/90 (5.6)	11/90 (12.2)	−6.6
Leidl et al. [24]	Uganda	Screened for LTBI	4/128 (3.1)	6/128 (4.7)	−1.6
Ling et al. [31]	South Africa	Symptomatic ptes	27/108 (25.0)	2/108 (2.0)	23.0
Dheda et al. [33]	South Africa	Symptomatic ptes	8/20 (40.0)	1/20 (5.0)	35.0
Latorre et al. [41]	Spain	Screened for LTBI	1/75 (1.3)	1/75 (1.3)	0
Dominguez et al. [46]	Spain	Screened for LTBI	0/19	0/19	–
Talati et al. [47]	USA	Screened for LTBI	6/336 (1.8)	47/336 (13.9)	−12.1

IGRAs: Interferon-γ release assays; QFT-GIT: QuantiFERON®-TB Gold In-Tube; n/N: indeterminate results/individuals tested; TB: tuberculosis; LTBI: latent tuberculosis infection; ptes: patients.

The pooled indeterminate rates for high-burden countries were 12.0% (95% CI 8.6–16.4; *I* ^2^ = 44.5%) for QFT-GIT and 7.7% (95% CI 3.6–15.5; *I* ^2^ = 47.6%) for T-SPOT.TB. Pooled indeterminate rates in low/intermediate-burden countries were 6.4% (95% CI 1.1–12.9; *I* ^2^ = 47.6%) for QFT-GIT and 3.5% (95% CI 1.4–8.4; *I* ^2^ = 44.8%) for T-SPOT.TB. When stratified for type of patients (patients evaluated because of suspicion of tuberculosis, patients with culture-confirmed tuberculosis and patients screened for LTBI), pooled indeterminate rates for QFT-GIT were higher for patients with active tuberculosis (15.3%; 95% CI 10.8–21.2; *I^2^* = 17.1%) and for symptomatic patients (12.3%; 95% CI 6.9–39.4; *I^2^* = 48.4%) than for those screened for LTBI (3.9%; 95% CI 2.4–6.4; *I^2^* = 45.3%). Pooled indeterminate rates for T-SPOT.TB were higher for symptomatic patients (9.1%; 95% CI 4.0–19.3; *I^2^* = 48.2%) than for those screened for LTBI (4.3%; 95% CI 2.2–8.1; *I^2^* = 42.9%).

**Figure 5:**
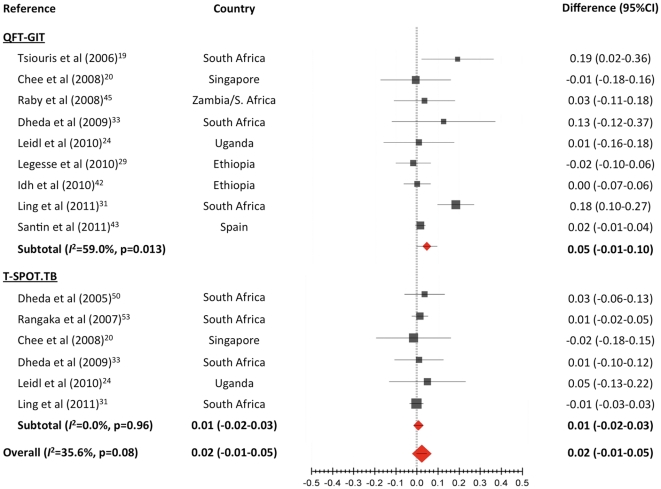
Comparison of the proportion of indeterminate results between HIV-infected and HIV-uninfected individuals. Pooled estimates derived from random effects (DerSimonian-Laird) modeling.

Eleven studies allowed a comparison of rates of indeterminate results between HIV-infected and HIV-uninfected individuals [19], [20], [24], [29], [31], [33], [42], [43], [45], [50], [53]. Indeterminate rates were higher in HIV-infected than in HIV-uninfected individuals for the QFT-GIT test, but the difference did not reach statistical significance (difference 4.6%; 95% CI -1 to 10; *I* ^2^ = 58.8%), as was the case of T-SPOT.TB (difference 0.7%; 95% CI -2 to 3; *I* ^2^ = 0.0%) (Figure 5).

#### Effect of CD4^+^ cell counts on indeterminate results

**Figure 6:**
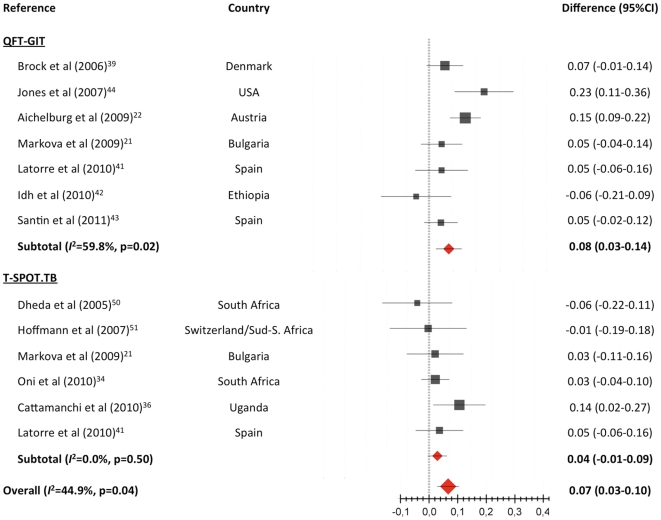
Differences in the proportion of indeterminate results of IGRAs by 200 CD4^+^ cell count threshold. Pooled estimates derived from random effects (DerSimonian-Laird) modeling.

Indeterminate result rates according to 200 CD4+ T-cell count threshold could be pooled from seven studies with QFT-GIT [21], [22], [39], [41]–[44] and six with T-SPOT.TB [21], [34], [36], [41], [50], [51]. The pooled indeterminate rates were 11.6% (95% CI 7.0–18.6; *I* ^2^ = 34.7%) for CD4+<200, and 3.1% (95% CI 1.1–8.5; *I* ^2^ = 47.4%) for CD4+ ≥200 (difference 8.1%; 95% CI 2.6–13.7; *I* ^2^ = 59.8%) for QFT-GIT, and 11.4% (95% CI 5.1–23.8; *I* ^2^ = 43.1%) for CD4+<200, and 7.9% (95% CI 4.6–13.3; *I* ^2^ = 24.6%) for CD4+≥200 (difference 4.0%; 95% CI -0.5 to 8.5; *I* ^2^ = 0.0%) for T-SPOT.TB (Figure 6).

## Discussion

This SR provides a comprehensive summary of the current evidence on the performance of the two commercial IFN-γ-based assays for the immunodiagnosis of tuberculosis and tuberculosis infection in HIV-infected adults. The main results can be summarized as follows. First, the sensitivity and specificity of either IGRA in HIV-infected people is suboptimal for being used alone to rule in or rule out active tuberculosis disease. Second, the risk of tuberculosis in the short- to medium-term in HIV-infected adults with a negative QFT-GIT seems to be low. Third, indeterminate results of IGRAs were more frequent in HIV-infected patients with active tuberculosis from high-burden tuberculosis countries. Fourth, HIV-associated immunosuppression, measured by circulating CD4^+^ T-lymphocytes, negatively affects the performance of QFT-GIT, and to a lesser extent, T-SPOT.TB.

The sensitivity of IGRAs for culture-confirmed tuberculosis in the current SR was lower than that reported in three meta-analyses including predominantly immunocompetent people [8], [9], [11], and similar to that reported for HIV-infected patients in the three previous SRs [10], [11], [12]. Taken together, the results of the previous SRs and our own show that the sensitivity of QFT-GIT is roughly 65%, ranging between 61% reported by Cattamanchi et al. [10] in low-income countries and 68% reported by Chen et al. [12] in both high and low-income countries. For T-SPOT.TB, the sensitivity was close to 70%, ranging between 65% obtained in the current SR and 72% reported by Cattamanchi et al. [10] in low-income countries. These figures mean that, at best, IGRAs will miss one in three cases of active tuberculosis (Table 7).

**Table 7:** Sensitivity and specificity of the IGRAs in HIV-infected patients in four systematic reviews.

	Cattamanchi	Metcalfe	Chen	
	(Ref. [10])	(Ref. [11])	(Ref. [12])	Current SR
**Sensitivity** *				
High-burden TB settings				
-QFT-GIT	61% (47–75)	65% (52–77)	N.D.	61% (53–69)
-T-SPOT.TB	72% (62–81)	68% (56–80)	N.D.	65% (54–74)
Low-burden TB settings				
-QFT-GIT	67% (47–83)†	N.D.	N.D.	59% (46–71)
-T-SPOT.TB	94% (73–100)†	N.D.	N.D.	69% (47–99)†
Overall				
-QFT-GIT	N.D.	N.D.	69% (62–71)	61% (54–67)
-T-SPOT	N.D.	N.D.	66% (60–71)	65% (56–74)
**Specificity**				
High-burden TB settings				
-QFT-GIT	N.D.	50% (35–65)	57% (54–60)	62% (49–74)
-T-SPOT.TB	N.D.	52% (40–63)	63% (58–68)	73% (54–85)
Low-burden TB settings				
-QFT-GIT	N.D.	N.D.	94% (93–96)	89%‡
-T-SPOT.TB	N.D.	N.D.	64% (44–81)†	64% (44–81)†
Overall				
-QFT-GIT				
-T-SPOT.TB	N.D.	N.D.	63%(58–68)	70% (55–83)

NOTE: Stratification for high-burden and low-burden TB settings is roughly equivalent to low-income and high-income settings used by Cattamanchi [10], Metcalfe [11] and Chen [12] in their systematic reviews; TB = tuberculosis; QFT-GIT = QuantiFERON Gold® In-Tube; N.D. = Not done.

* Sensitivity estimated by scoring indeterminate results negative;

† Only one study;

‡ Two studies: 89% each respectively.

HIV-associated immunosuppression, measured by circulating CD4^+^ T-cells, weakens the ability of IGRAs to detect tuberculosis infection. A previous SR [10] explored the impact of immunosuppression on the proportion of positive results according to a 200 CD4^+^ T-cell threshold, regardless of whether they had active tuberculosis or not. However, the value of the information provided by this approach is limited because the analysis included healthy people with unknown LTBI status. In the current SR, we tried to determine the impact of CD4^+^ T-cell counts on the sensitivity of IGRAs in HIV-infected patients with active tuberculosis disease, but the results were inconclusive. While one of the three studies with QFT-GIT [23], [28], [31] observed lower sensitivity with CD4^+^ below 200 cells/mm^3^ [23], another one found higher sensitivity with CD4^+^ below 200 cells/mm^3^ [31], and a third one did not find significant differences in CD4^+^ T-cell counts between patients with either positive or negative QFT-GIT [28]. As for T-SPOT.TB, while two of the three studies [31], [34], [37] found no change in sensitivity with CD4+ T-cell counts [34], [37], the other one found higher sensitivity in patients with CD4^+^ below 200 cells/mm^3^ [31]. Since the decrease in sensitivity of IGRAs in HIV-infected patients is largely due to high rates of indeterminate results, the correct reporting of these results is essential for an accurate assessment of the sensitivity of the IGRA tests. Unfortunately, indeterminate results due either to a high-background production of interferon-γ (negative control) or to a failure test due to an insufficient number of PBMCs are often explicitly excluded or not reported. In fact, in the three studies that provided these data, a third of all invalid T-SPOT.TB results were due to failed T-SPOT.TB tests because of a lack of cells [36], [37], [47]. This may lead to an overestimation of the sensitivity of T-SPOT.TB assay in HIV-infected patients, and challenges the commonly held assumption that performance of T-SPOT.TB is less affected (if at all) by CD4^+^ T-cell depletion than QFT-GIT.

It has been suggested that IGRAs are less affected than TST by HIV-associated immunosuppression. However, there is no consistent evidence that the IGRAs are more sensitive for detecting tuberculosis infection in patients with active disease. Data from the five studies reporting comparisons between QFT-GIT and TST yielded a pooled sensitivity of 67% and 60% respectively. Actually, in the largest study, which included more than 800 patients, TST was at least as sensitive as QFT-GIT [32].

As might be expected, the specificity of either IGRA for active tuberculosis disease was suboptimal for use as a rule-in test [9]. Although IGRAs use highly *M. tuberculosis*-specific antigens, since they do not distinguish between latent and active infection they cannot provide optimal specificity. Besides, they reflect the high prevalence of LTBI in the countries in which most of the studies were conducted [57]. Whether the specificity of IGRAs in low-burden tuberculosis settings is better is currently unclear. The present SR identified only three studies in low-burden settings, all from Italy: two with QFT-GIT [26], [27] and one with T-SPOT.TB [38]. Specificity was 89% for QFT-GIT in both studies, and 64% for T-SPOT.TB (Table 7).

Although culture-confirmed tuberculosis has been commonly used as a surrogate for tuberculosis infection, tuberculosis-associated immunodeficiency may impair the ability of IGRAs to detect the infection, particularly in HIV-infected patients. Therefore, their actual sensitivity for LTBI may be underestimated by extrapolating from patients with active disease [58]. Determining the capability of IGRAs to predict the risk of subsequent active tuberculosis is another way of evaluating the IGRAs suitability for detecting LTBI. A comprehensive SR, including mainly studies with non-HIV-infected individuals, showed a marginal advantage of IGRAs over the TST for predicting incident active tuberculosis [59]. Two studies identified in the current SR, both conducted in low-burden tuberculosis countries, showed modest associations between positive IGRA result and incident active tuberculosis in the short- to medium term [22], [52]. Conversely, a negative result of QFT-GIT had a high negative predictive value (100%) in two studies [22], [43]. These data, if further confirmed in large, longitudinal and properly designed studies, would help to improve the targeting of at-risk patients by reducing the number of people considered for preventive treatment.

Indeterminate results, due either to low IFN-γ production in the positive control or to high IFN-γ production in the negative control, may negatively affect the overall utility of IGRAs. The proportion of indeterminate results in the current SR showed huge differences across studies, ranging from no indeterminate results at all to rates as high as 25% and 33% for QFT-GIT and T-SPOT.TB respectively [31], [36]. These differences are related to host characteristics (CD4^+^ cell counts), type of evaluated people (patients with active tuberculosis vs. people evaluated for LTBI), and setting (high-burden and resource-limited vs. low-burden and high-income settings), but are also due to differences in the criteria used for reporting data. Indeterminate results due to high-background IFN-γ production, as well as failure T-SPOT.TB tests due to an insufficient number of PBMCs, are often not counted as such and are excluded from the analyses. Therefore, the calculation of indeterminate result rates and their association with potentially influencing factors will inevitably be compromised by these limitations. Interestingly, the types of indeterminate results were not equally distributed for the two assays. While low IFN-γ production upon stimulation with PHA (positive control) accounted for more than 90% of the indeterminate results with the QFT-GIT assay, half of the indeterminate T-SPOT.TB assays were due to high-background IFN-γ production (negative control).

The pooled indeterminate rates for the two assays were higher in high-burden settings than in low-burden settings. They were also higher in patients with symptoms suggestive of tuberculosis or culture-confirmed tuberculosis than in those screened for LTBI. Because studies that enrolled patients with active tuberculosis were mainly carried out in high-burden countries, whilst those that enrolled patients screened for LTBI were from low-burden countries, further analyses to determine which of the two factors has a greater influence on the occurrence of indeterminate results cannot be performed. On the one hand, HIV-infected patients usually have profound CD4^+^ T-lymphocyte depletion either as a cause or as a consequence of the disease, which may cause anergy and indeterminate IGRA results [58]. On the other hand, indeterminate results have been related to operational factors mainly linked to resource-limited settings, such as delayed incubation [60]–[62], and the location of the laboratory at which the samples are processed (according to data from Zambia; K. Shanaube, personal communication) (Table 8).

**Table 8:** Proportion of indeterminate results of IGRAs in HIV-infected patients in four systematic reviews.

	Cattamanchi	Metcalfe	Chen	
	(Ref. [10])	(Ref. [11])	(Ref. [12])	Current SR
**Setting**				
High-burden TB				
-QFT-GIT	4% (1–9)*	15% (9–21)†	11.4% (9.7–13.2)†	12.0% (8.6–16.4)
-T-SPOT.TB	2% (0–3)*	9% (0–17)†	14% (11.4–17.1)†	7.7% (3.6–15.5)
Low-burden TB				
-QFT-GIT	4% (3–6)*	N.D.	8.4% (6.8–10.2)†	6.4% (1.1–12.9)
-T-SPOT.TB	5% (1–9)*	N.D.	0% (0–0.9)†	3.5% (1.4–8.4)
**Population evaluated**				
Active TB				
-QFT-GIT	N.D.	N.D.	N.D.	15.3% (10.8–21.2)
-T-SPOT.TB	N.D.	N.D.	N.D.	9.1% (4.0–19.3)
Screened for LTBI				
-QFT-GIT	N.D.	N.D.	N.D.	3.9% (2.4–6.4)
-T-SPOT.TB	N.D.	N.D.	N.D.	4.3% (2.2–8.1)
**Difference by 200 CD4+ count threshold**				
-QFT-GIT	N.D.	N.D.	N.D.	8.1% (2.6–13.7)
-T-SPOT.TB	N.D.	N.D.	N.D.	4.0% (−0.5 to 8.5)
**Overall**				
-QFT-GIT	N.D.	N.D.	10.0% (8.8–11.3)	8.2% (6.0–11.2)
-T-SPOT.TB	N.D.	N.D.	13.2% (10.6–16.0)	5.9% (3.5–9.8)

NOTE: Stratification for high-burden and low-burden TB settings is roughly equivalent to low-income and high-income settings used by Cattamanchi [10], Metcalfe [11] and Chen [12] in their systematic reviews; TB = tuberculosis; LTBI = latent tuberculosis infection; QFT-GIT = QuantiFERON Gold® In-Tube; N.D. = Not done.

* Only patients screened for LTBI included;

† Symptomatic patients, with and without active TB.

Our SR has limitations. First, the validity of the results is limited by the inconsistency across the studies. This heterogeneity persisted after performing subgroup analyses. Second, the main body of literature on active tuberculosis comes from high-burden tuberculosis and resource-limited settings, which limits the generalization of our estimates. Conversely, studies for the prediction of subsequent development of active disease in HIV-infected patients were exclusively from low-burden countries. Therefore, the low risk of subsequent active tuberculosis for patients testing negative on QFT-GIT obtained in two low-burden countries in Europe cannot be extrapolated to countries with high burdens of tuberculosis such as Sub-Saharan African countries. Finally, the lack of an adequate standard for latent tuberculosis infection is a inherent limitation to every SR to draw confident estimates on the capacity of IGRA tests to detect tuberculosis infection in people without evidence of active disease.

Nonetheless, some relevant conclusions may be drawn from this SR. First, the current evidence indicates that neither IGRA is able to replace conventional microbiological diagnosis of tuberculosis in HIV-infected patients. Second, QFT-GIT, if the low risk of subsequent active tuberculosis in HIV-infected patients testing negative is confirmed, could replace TST for targeting at-risk patients for chemoprophylaxis in low-burden tuberculosis countries. Third, potential causes of invalid tests, such as delayed incubation and other operational factors, should be addressed in order to improve the performance of IGRAs, particularly in resource-limited high-burden tuberculosis countries with high HIV-coinfection prevalence.

## References

[1] Giraldi E, Antonucci G, Vanacore P, Palmieri F, Matteelli A (2004). Tuberculosis in HIV-infected persons in the context of wide availability of highly active antiretroviral therapy.. Eur Respir J.

[2] World Health Organization website.. http://whqlibdoc.who.int/publications/2011/9789241564380_eng.pdf.

[3] Centers for Disease Control and Prevention (2009). Guidelines for prevention and treatment of opportunistic infections in HIV-infected adults and adolescents.. MMWR Early Release.

[4] Jones BE, Young SM, Antoniskis D, Davidson PT, Kramer F (1993). Relationship of the manifestations of tuberculosis to CD4 cell counts in patients with human immunodeficiency virus infection.. Am Rev Respir Dis.

[5] Markowitz N, Hansen NI, Wilcosky TC, Hopewell PC, Glassroth J (1993). Tuberculin and anergy testing in HIV seropositive and HIV seronegative persons. Pulmonary complications of HIV infection study group.. Ann Intern Med.

[6] Santin-Cerezales M, Dominguez-Benitez J (2011). Diagnosis of tuberculosis infection using interferon-γ-based assays.. Enferm Infecc Microbiol Clin.

[7] Pai M, Zwerling A, Menzies D (2008). Systematic review: T-cell-based assays for the diagnosis of latent tuberculosis infection: an update.. Ann Intern Med.

[8] Diel R, Loddenkemper R, Nienhaus A (2010). Evidence-based comparison of commercial interferon-γ release assays for detecting active TB. A metaanalysis.. Chest.

[9] Sester M, Sotgiu G, Lange C, Giehl C, Girardi E (2011). Interferon-γ release assays for the diagnosis of active tuberculosis: a systematic review and meta-analysis.. Eur Respir J.

[10] Cattamanchi A, Smith R, Steingart KR, Metcalfe JZ, Date A (2011). Interferon-gamma release assays for the diagnosis of latent tuberculosis infection in HIV-infected individuals: a systematic review and meta-analysis.. J Acquir Immune Defic Syndr.

[11] Metcalfe JZ, Everett CK, Steingart KR, Cattamanchi A, Huang L (2011). Interferon-γ release assays for active pulmonary tuberculosis diagnosis in adults in low- and middle-income countries: systematic review and meta-analysis.. Clin Infect Dis.

[12] Chen J, Zhang R, Wang J, Liu L, Zheng Y (2011). Interferon-gamma release assays for the diagnosis of active tuberculosis in HIV-infected patients: a systematic review and meta-analysis.. PLoS ONE.

[13] World Health Organization website.. http://whqlibdoc.who.int/hq/2011/WHO_HTM_TB_2011.17_eng.pdf.

[14] Moher D, Liberati A, Tetzlaff J, Altman DG, and the PRISMA Group (2009). Preferred reporting items for systematic reviews and meta-analyses: The PRISMA statement.. PLoS Med.

[15] Whiting PF, Weswood ME, Rutjes AWS, Reitsma JB, Bossuyt PNM (2006). Evaluation of QUADAS, a tool for the quality assessment of diagnostic accuracy studies.. BMC Med Res Methodol.

[16] Wells GA, Shea B, O'Connell D, Peterson J, Weich V The Newcasttle-Ottawa Scale (NOS) for assessing the quality of nonrandomised studies in meta-analyses.. http://www.ohri.ca/programs/clinical_epidemiology/oxford.htm.

[17] World Health Organization website.. http://www.who.int/tb/country/data/profiles/en/index.html.

[18] Wallace BC, Schmid CH, Lau J, Trikalinos TA (2009). Meta-analyst: software for meta-analysis of binary, continuous and diagnostic data.. BMC Med Res Methodol.

[19] Tsiouris SJ, Coetzee D, Toro PL, Austin J, Stein Z (2006). Sensitivity analysis and potential uses of a novel gamma interferon release assay for diagnosis of tuberculosis.. J Clin Microbiol.

[20] Chee CB, Gan SH, Khinmar KW, Barkham TM, Koh CK (2008). Comparison of sensitivities of two commercial gamma interferon release assays for pulmonary tuberculosis.. J Clin Microbiol.

[21] Markova R, Todorova Y, Drenska R, Elenkov I, Yankova M (2009). Usefulness of interferon-gamma release assays in the diagnosis of tuberculosis infection in HIV-infected patients in Bulgaria.. Biotechnol Biotechnol Equip.

[22] Aichelburg MC, Rieger A, Breitenecker F, Pfistershammer K, Tittes J (2009). Detection and prediction of active tuberculosis disease by a whole-blood interferon-gamma release assay in HIV-1-infected individuals.. Clin Infect Dis.

[23] Kabeer BSA, Sikhamani R, Swaminathan S, Perumal V, Paramasivam P (2009). Role of interferon gamma release assay in active TB diagnosis among HIV infected individuals.. PLoS One.

[24] Leidl L, Mayanja-Kizza H, Sotgiu G, Baseke J, Ernst M (2010). Relationship of immunodiagnostic assays for tuberculosis and numbers of circulating CD4+ T-cells in HIV infection.. Eur Resp J.

[25] Garcia-Gasalla M, Fernández-Baca V, Mir Viladrich I, Cifuentes-Luna C, Campins-Roselló A (2010). Quantiferon-TB-Gold In-Tube test in the diagnosis of pulmonary and extra-pulmonary tuberculosis.. Enferm Infecc Microbiol Clin.

[26] Sauzullo I, Mengoni F, Scribo R, Valesini G, Potenza C (2010). Evaluation of QuantiFERON-TB Gold In-Tube in human immunodeficiency virus infection and in patient candidates for anti-tumour necrosis factor-alpha treatment.. Int J Tuberc Lung Dis.

[27] Bua A, Molicotti P, Ruggeri M, Madeddu G, Ferrandu G (2011). Interferon-γ release assay in people infected with human immunodeficiency virus.. Clin Microbiol Infect.

[28] Kabeer BS, Sikhamani R, Raja A (2010). Comparison of interferon gamma and interferon gamma-inducible protein-10 secretion in HIV-tuberculosis patients.. AIDS.

[29] Legesse M, Ameni G, Mamo G, Medhin G, Bjune G (2010). Performance of QuantiFERON-TB Gold In-Tube (QFTGIT) for the diagnosis of *Mycobacterium tuberculosis* (Mtb) infection in Afar Pastoralists, Ethiopia.. BMC Infect Dis.

[30] Veldsman C, Kock MM, Rossouw T, Nieuwoudt M, Maeurer M (2009). QuantiFERON-TB GOLD ELISA assay for the detection of *Mycobacterium tuberculosis*-specific antigens in blood specimens of HIV-positive patients in a high-burden country.. FEMS Immunol Med Microbiol.

[31] Ling DI, Pai M, Davids V, Brunet L, Lenders L (2011). Are interferon-γ release assays useful for active tuberculosis in a high-burden setting?. Eur Respir J.

[32] Rangaka MX, Gideon HP, Wilkinson KA, Pai M, Mwansa-Kambafilwe J (2012). No discriminatory value of interferon release added to smear negative HIV-tuberculosis algorithms.. Eur Respir J.

[33] Dheda K, van Zyl-Smit RN, Meldau R, Symons G, Khalfey H (2009). Quantitative lung T cell responses aid the rapid diagnosis of pulmonary tuberculosis.. Thorax.

[34] Oni T, Patel J, Gideon HP, Seldon R, Wood K (2010). Enhanced diagnosis of HIV-associated tuberculosis by relating T-SPOT.TB and CD4 counts.. Eur Resp J.

[35] Tan CK, Hung CC, Lai CC, Liao CH, Chou CH (2010). Diagnosis of active tuberculosis by enzyme-linked immunospot assay for interferon-gamma in HIV-infected patients.. J Acquir Immune Defic Syndr.

[36] Cattamanchi A, Ssewenyana I, Davis JL, Huang L, Worodria W (2010). Role of interferon-gamma release assays in the diagnosis of pulmonary tuberculosis in patients with advanced HIV infection.. BMC Infect Dis.

[37] Chen J, Sun J, Zhang R, Liu L, Zheng Y (2011). T-SPOT.TB in the diagnosis of active tuberculosis among HIV-infected patients with advanced immunodeficiency.. AIDS Res Hum Retroviruses.

[38] Vincenti D, Carrara S, Butera O, Bizzoni F, Casetti R (2007). Response to region of difference 1 (RD1) epitopes in human immunodeficiency virus (HIV)-infected individuals enrolled with suspected active tuberculosis: a pilot study.. Clin Exp Immunol.

[39] Brock I, Ruhwald M, Lundgren B, Westh H, Mathiesen LR (2006). Latent tuberculosis in HIV positive, diagnosed by the *M. tuberculosis* specific interferon-gamma test.. Respir Research.

[40] Luetkemeyer AF, Charlebois ED, Flores LL, Bangsberg DR, Deeks SG (2007). Comparison of an interferon-gamma release assay with tuberculin skin testing in HIV-infected individuals.. Am J Respir Crit Care Med.

[41] Latorre I, Martinez-Lacasa X, Font R, Lacoma A, Puig J (2010). IFN-γ response on T-cell based assays in HIV-infected patients for detection of tuberculosis infection.. BMC Infect Dis.

[42] Idh J, Abate E, Westman A, Elias D, Janols H (2010). Kinetics of the QuantiFERON-TB Gold In-Tube test during treatment of patients with sputum smear-positive tuberculosis in relation to initial TST result and severity of disease.. Scand J Infect Dis.

[43] Santin M, Casas S, Saumoy M, Andreu A, Moure R (2011). Detection of latent tuberculosis by the tuberculin skin test and a whole-blood interferon-γ release assay, and the development of active tuberculosis in HIV-seropositive persons.. Diagn Clin Microbiol Infect Dis.

[44] Jones S, de Gijsel D, Wallach FR, Gurtman AC, Shi Q (2007). Utility of QuantiFERON-TB Gold In-Tube testing for latent TB infection in HIV-infected individuals.. Int J Tuberc Lung Dis.

[45] Raby E, Moyo M, Devendra A, Banda J, De Haas P (2008). The effects of HIV on the sensitivity of a whole blood IFN-gamma release assay in Zambian adults with active tuberculosis.. PLoS ONE.

[46] Domínguez J, Ruiz-Manzano J, De Souza-Galvão M, Latorre I, Milà C (2008). Comparison of two commercially available gamma interferon blood tests for immunodiagnosis of tuberculosis.. Clin Vaccine Immunol.

[47] Talati NJ, Seybold U, Humphrey B, Aina A, Tapia J (2009). Poor concordance between interferon-gamma release assays and tuberculin skin tests in diagnosis of latent tuberculosis infection among HIV-infected individuals.. BMC Infect Dis.

[48] Richeldi L, Losi M, D'Amico R, Luppi M, Ferrari A (2009). Performance of tests for latent tuberculosis in different groups of immunocompromised patients.. Chest.

[49] Shanaube K, Hargreavesn J, Fielding K, Schaap A, Lawrence KA (2011). Risk factors associated with positive QuantiFERON-TB Gold In-Tube and tuberculin skin tests results in Zambia and South Africa.. PLoS One.

[50] Dheda K, Lalvani A, Miller RF, Scott G, Booth H (2005). Performance of a T-cell-based diagnostic test for tuberculosis infection in HIV-infected individuals is independent of CD4 cell count.. AIDS.

[51] Hoffmann M, Reichmuth M, Fantelli K, Schoch OD, Fierz W (2007). Conventional tuberculin skin testing versus T-cell-based assays in the diagnosis of latent tuberculosis infection in HIV-positive patients.. AIDS.

[52] Clark SA, Martin SL, Pozniak A, Steel A, Ward B (2007). Tuberculosis antigen-specific immune responses can be detected using enzyme-linked immunospot technology in human immunodeficiency virus (HIV)-1 patients with advanced disease.. Clin Exp Immunol.

[53] Rangaka MX, Wilkinson KA, Seldon R, Van Cutsem G, Meintjes GA (2007). Effect of HIV-1 infection on T-cell-based and skin test detection of tuberculosis infection.. Am J Respir Crit Care Med.

[54] Mandalakas AM, Hesseling AC, Chegou NN, Kirchner HL, Zhu X (2008). High level of discordant IGRA results in HIV-infected adults and children.. Int J Tuberc Lung Dis.

[55] Stephan C, Wolf T, Goetsch U, Bellinger O, Nisius G (2008). Comparing QuantiFERON-tuberculosis gold, T-SPOT tuberculosis and tuberculin skin test in HIV-infected individuals from a low prevalence tuberculosis country.. AIDS.

[56] Rivas I, Latorre I, Sanvisens A, Dominguez J, Tort J (2009). Prospective evaluation of latent tuberculosis with interferon-gamma release assays in drug and alcohol abusers.. Epidemiol Infect.

[57] Barth RE, Mudrikova T, Hoepelman AI (2008). Interferon-gamma release assays (IGRAs) in high-endemic settings: could they play a role in optimizing global TB diagnostics? Evaluating the possibilities of using IGRAs to diagnose active TB in a rural African setting.. Int J Infect Dis.

[58] Hougardy JM, Place S, Hildebrand M, Drowart A, Debrie AS (2007). Regulatory T Cells depress immune responses to protective antigens in active tuberculosis.. Am J Respir Crit Care Med.

[59] Rangaka MX, Wilkinson KA, Glynn JR, Ling D, Menzies D (2012). Predictive value of interferon-γ release assays for incident active tuberculosis: a systematic review and meta-analysis.. Lancet Infect Dis.

[60] Shanaube K, De Haas P, Schaap A, Moyo M, Kosloff B (2010). Intra-assay reliability and robustness of QuantiFERON®-TB Gold In-Tube test in Zambia.. Int J Tuberc Lung Dis.

[61] Herrera V, Yeh E, Murphy K, Parsonnet J, Banaei N (2010). Immediate incubation reduces indeterminate results for QuantiFERON-TB Gold In-Tube assay.. J Clin Microbiol.

[62] Doberne D, Gaur RL, Banaei N (2011). Preanalytical delay reduces sensitivity of QuantiFERON-TB Gold In-Tube assay for detection of latent tuberculosis infection.. J Clin Microbiol.

